# Optimizing multimodal scene recognition through relevant feature selection approach for scene classification

**DOI:** 10.1016/j.mex.2025.103226

**Published:** 2025-02-17

**Authors:** Sumathi K, Pramod Kumar S, H R Mahadevaswamy, Ujwala B S

**Affiliations:** a JNN College of Engineering, Shimoga, Karnataka, India; b JSS Mahavidyapeetha, Visvesvaraya Technological University, Mysore, Karnataka, India

**Keywords:** Mutual information, Feature extraction, Scene classification, Multimodal Feature extraction and Relevant Feature selection using Filter and Embedded approach

## Abstract

Scene classification plays a vital role in various computer vision applications, but building deep learning models from scratch is a very time-intensive process. Transfer learning is an excellent classification method using the predefined model. In our proposed work, we introduce a novel method of multimodal feature extraction and a feature selection technique to improve the efficiency of transfer learning in scene classification. We leverage widely used convolutional neural networks (CNN) for feature extraction, followed by relevant feature selection techniques to enhance the performance of the model and increase computational efficiency. In this work, we have executed the proposed method on the Scene dataset of 6 classes and the AID dataset. Experimental results indicate that the MIFS-based approach reduces computational overhead and achieves competitive or superior classification accuracy. The proposed methodology offers a scalable and effective solution for scene classification tasks, with potential applications in real-time recognition and automated systems.

## Graphical abstract

**Figure:**
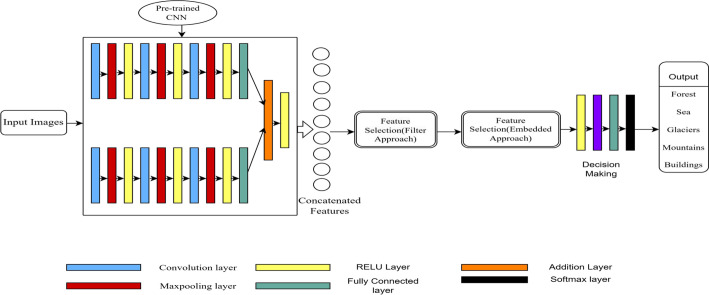

Specifications Table

**Table:** 

Subject area:	Computer Science
More specific subject area:	Deep Learning
Name of your method:	Multimodal Feature extraction and Relevant Feature selection using Filter and Embedded approach.
Name and reference of original method:	Agrawal, C., Pandey, A. and Goyal, S., 2024. Multimodal fake news detection using hyperparameter-tuned BERT and ResNet110. *International Journal of Advanced Technology and Engineering Exploration, 11*(114), p.759.
Resource availability:	NA

## Background

Scene recognition is a pivotal task that covers a wide range of applications, from autonomous vehicle navigation systems to image management systems. In our proposed methodology, we have employed the novel technique of scene recognition using CNN combined with multimodal deep learning methodologies and a feature selection method to extract suitable features and remove redundant features. The scene dataset employed in our experimentation has six different classes of images. For example, ResNet50, based on the ResNet architecture, is 50 layers deep and is designed to achieve higher classification accuracy for complex tasks by utilizing deeper layers [1]. It leverages the knowledge learned from large-scale datasets in related tasks, such as image classification, and transfers it to the scene recognition task, which may have limited data availability [2]. Incorporating transfer learning with pre-trained convolutional neural networks (CNNs) has obtained popularity for classifying small image datasets.

Models are pre-trained on large datasets like ImageNet and are commonly used as feature extractors, greatly reducing the number of parameters requiring training. These extracted features are subsequently passed to a classifier for training and prediction. In addition to mitigating overfitting, classification success depends on the quality and optimal selection of the extracted feature set. Scene recognition traditionally relies on an unimodal approach, where a single model analyzes image or video inputs. Multimodal deep learning techniques have shown good performance in scene classification, overcoming the limitations of traditional unimodal scene recognition approaches. This paper focuses on employing multimodal deep learning techniques, intending to leverage diverse modalities to enhance the accuracy and robustness of scene interpretation. Our methodology centers on the innovative integration of multiple modalities into a unified input for scene recognition, offering a distinct departure from conventional models that typically process data from only one modality. By employing multimodal deep learning on a single input image, this approach enables the simultaneous analysis of multiple data forms, such as text, audio, and depth information all derived from the same image. In this work, we have incorporated various modalities into one model, leveraging their complementary characteristics to understand the scene comprehensively. We apply the feature selection technique to refine the quality of feature representations obtained through this fusion. This technique enhances the overall feature quality, discriminative power, and robustness, yielding substantial benefits for scene identification tasks. In our model, the Softmax layer performs the final recognition by considering the inputs from the multimodal network to assign the scene labels. The approach detailed in this study optimizes the advantages of multimodal fusion, resulting in significant enhancements in both accuracy and robustness for scene recognition. According to the survey to recognise the multiple scenes, Hua et al. [3] have developed a prototype network by considering the several annotated images for each instance. This network has three blocks: a prototype learning module, a multi-head attention memory retrieval module, and an external memory to store the prototypes. Using the AID dataset, an F1 score of 57.40 % was achieved. Petrovska et al. [4] have introduced a technique for extracting features from the fine-tuned CNNs and utilizing features for recognizing the remote sensing images using a Support Vector Machine using both linear and radial basis kernel functions and also introduced a transfer learning to fine-tune pre-trained Convolutional Neural Networks (CNNs) in an end-to-end manner. To reduce overfitting, the label smoothing regularization technique is incorporated into their approach. They use inception- and residual-based CNNs such as Inception-v3, Xception, ResNet50, and DenseNet121 for fine-tuning and feature extraction, achieving up to 98 % classification accuracy, surpassing other methods. Wang et al. [5] introduced a neural network for aerial scene classification that combines a depth-wise separable convolution (CSDS) architecture with a channel-spatial attention mechanism. They constructed the DS-Conv and pyramid residual connection architectures, efficiently recovering and merging channel features and reducing computational demands. The pyramid residual connections link features across layers to capture relationships. To minimize the impact of related categories, the cross-entropy loss function is used during backpropagation, achieving an accuracy of 94.29 % on the AID dataset. Zhao et al. [6] integrated a channel-spatial attention mechanism into a residual dense network framework for remote sensing image classification. They efficiently combine multilayer convolutional features using residual dense blocks, while the channel-spatial attention module enhances feature representation. After applying data augmentation, scenes are classified using a SoftMax classifier, achieving an accuracy of 94.15 % on the AID dataset. Bazi et al. [6] introduced a remote sensing image classification technique based on vision transformers, where images are partitioned into smaller segments, then flattened and embedded in sequence. This sequence is passed through multiple multi-headattention layers to generate the final representation, with the initial token sequence fed into a SoftMax layer for classification [7].

## Method details

**Fig. 1:**
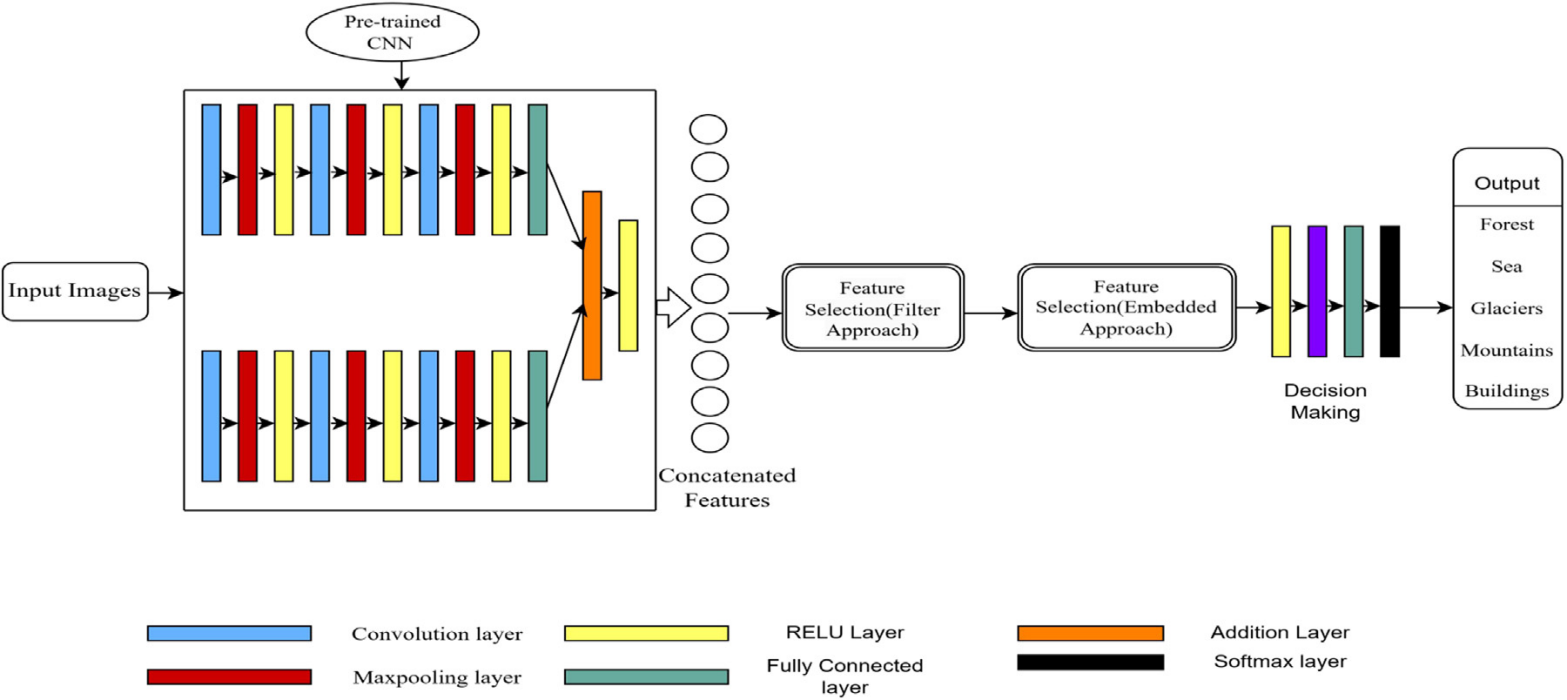
General block diagram of our multimodal network with feature selection approach.

Our proposed methodology consists of feature extraction and feature selection. Our method focuses on single-input multi-model fusion of scene recognition, where scene images are given as input to the multi-model network. Feature extraction is performed by combining the discriminative features of input data. Block diagram of the proposed methodology is shown in Fig. 1. Here, we have employed a transfer learning methodology for the scene recognition task, which increases the system's efficiency. In this method, features are obtained from the fully connected layer and merged through the addition layer incorporated in the model. The final recognition of the scene images is obtained by integrating the softmax activation function. After concatenating the features obtained by the multimodal network using pre-trained CNN, the feature selection technique uses a filter and embedded approaches to select the appropriate features. The feature selection technique mainly focuses on determining the relevant features and discarding the irrelevant or redundant features from the concatenated feature data. This feature set is subsequently used for Scene classification.

The above block diagram in Fig. 1 shows the proposed method for scene recognition. It includes the convolution and pooling layers followed by a ReLU activation function, which helps in feature extraction, and selecting the important feature helps reduce the vector's dimension to be processed. As shown in the block diagram, the proposed feature selection methods are applied one after the other. The feature selection methods will help reduce redundant features and increase the system's performance. Table 1 summarizes the layers in our model.

**Table 1:** Deep learning model layers used in our experimentation.

Layer	Dimension	Operation	Parameters
Input Images of Scenes 200 × 200 × 3	200 × 200 × 3	Image Input	—
Convolution layer 1 1283 × 3 × 3 stride [1 1] with padding enabled	200 × 200 × 128	Convolution operation	Weights 3 × 3 × 3 × 128 Biases 1 × 1 × 128
Max pooling layer 1 with padding	200 × 200 × 128	Max Pooling 2 × 2	—
Relu activation function 1	200 × 200 × 128	ReLU	—
Convolution layer 3 643 × 3 × 128 with a stride [1 1] and padding enabled	200 × 200 × 64	Convolution	Weights= 3 × 3 x 128 × 64 Biases 1 × 1 × 64
Max pooling layer 3 2 × 2 with padding	200 × 200 × 64	Max Pooling	—
Relu activation function 3	200 × 200 × 64	ReLU	
Convolution layer 5 325 × 3 × 64 with stride [1 1] and padding enabled	200 × 200 × 32	Convolution	Weights = 3 × 3 × 128 × 32 Biases =1 × 1 × 32
Max pooling layer 5 2 × 2 with padding	200 × 200 × 32	Max Pooling	—
Relu activation function 5	200 × 200 × 32	ReLU	—
Fully Connected Layer 1 1024	1 × 1 × 1024	Fully Connected Layer	Weights = 1024 × 1280,000 Biases = 1024 × 1
Convolution layer 2: 1283 Filters with size 3 × 3 with a stride [1 1] and padding enabled	200 × 200 × 128	Convolution	Weights = 3 × 3 × 3 × 128 Biases= 1 × 1 × 128
Max pooling layer 2 2 × 2 with padding	200 × 200 × 128	Max Pooling	—
Relu activation function 2	200 × 200 × 128	ReLU	—
Convolution layer 4: 643 filters of size 3 × 128 with stride [1 1] and padding enabled	200 × 200 × 64	Convolution	Weights=3 × 3 x 128 × 64 Biases 1 × 1 × 64
Max pooling layer 4 2 × 2 with padding	200 × 200 × 64	Max Pooling	—
Relu activation function 4	200 × 200 × 64	ReLU	—
Convolution layer 6: 323 filters of size 3 × 64 with stride [1 1] and padding enabled	200 × 200 × 32	Convolution	Weights 3 × 3 × 64 × 32 Biases 1 × 1 × 32
Maxpool layer 6 2 *×* 2 with padding	200 × 200 × 32	Max Pooling	—
Relu activation function 6	200 × 200 × 32	ReLU	—
Fully Connected 2 1024	1 × 1 × 1024	Fully Connected	Weights:1024 × 1280,000 Biases: 1024 × 1
Element-wise addition for two inputs	1 × 1 × 1024	Addition	
Relu activation function 7	1 × 1 × 1024	ReLU	
Feature Selection Layer: 600 neurons in a fully connected configuration	1 × 1 × 600	Fully Connected	Weights: 600 *×* 1024 Bias: 600 *×* 1
Relu activation function_8	1 × 1 × 600	ReLU	
Dropout 50 % dropout	1 × 1 × 600	Dropout	
Fully Connected Layer 3	1 × 1 × 6	Fully Connected	Weights: 6 x 600 Bias: 6 × 1
Softmax activation	1 × 1 × 6	Softmax	
Classoutput Cross entropy loss		Output	

The main aspect of this architecture is its two-part feature extraction methods, which enable parallel data processing. The model captures the information by aggregating the features from two models, which improves scene recognition or classification capability. The addition layer combines the features from two Pathways. The output of the addition layer is applied as an input to the ReLU activation function to enhance the fusion. Next, a 'Feature Selection Layer' introduces a crucial step for feature selection. Positioned after the ReLU activation, this layer employs a fully connected layer has 600 units to identify the most appropriate features from the fused representation [8]. In this architecture, we have used Adam optimizer and defined a batch size of 4. Define the set of maximum epochs and adjust the learning rate. All the hyperparameters used in our explanation are detailed in Algorithm 1, Table 2.

**Table 2:** Hyperparameters used in our methodology.

Hyperparameter	Operation
Optimizer	Adam optimizer
Batch size	4
Kernel size	3 × 3 for Convolution operation 2 × 2 for Max Pooling operation
Number of kernels	Convolution layer1 and Convolutional layer2 =128 Filters Convolution layer3 and Convolution layer4 =64Filters Convolution layer5 and Convolution layer6 =32Filters
Nodes in Fully Connected layers	Fully Connected 1 and Fully Connected layer 2 = 1024 nodes each Fully Connected 3 = 6 nodes
Learning rate	1.00 × 10–4
Epochs	10
Learning rate schedule	Adaptive
Loss function	Cross Entropy
Validation Frequency	87

## Feature selection

Feature selection is a technique that minimizes the redundant features and selects the relevant features from the final set of concatenated features. Removing the redundant features helps reduce the number of parameters and prevents overfitting. In this picture selection process, we remove the features that do not have valuable information and help reduce the total number of parameters. Our methodology uses a filter approach to minimize redundant features and select the relevant features using an embedded approach. Mutual information between two features represents information about one feature that can be inferred from the other, essentially serving as a measure of dependence between them. Features with lower mutual information are chosen with different features in the feature set to reduce redundancy [9]. Here, the feature set consists of concatenated features. Mutual information, calculated based on entropy, follows a basic formula analogous to set theory in mathematics, as shown below. In this context, H(X) and H(Y) represent the marginal entropies of feature X and feature Y, respectively, and H(X, Y) represents the joint entropy between two features. A feature's entropy measures the amount of information it holds, where each feature is defined as a vector [10]. Entropy is calculated as the summation of $P\text{lo}g_{2}$ where *P* represents the probability of each element in the feature vector. A histogram is created to determine the likelihood of each unique component. However, in a deep neural network, each feature corresponds to a single value representing the output of a neuron, and its probability is calculated using the softmax activation function. In the formula, if H(X, Y)=0, indicating zero joint probability, then X and Y are independent, meaning there is no mutual information, so I(X, Y)=0. This concept is incorporated into our proposed method.

### Feature set relevancy maximization

Features with lower mutual dependence are deemed more relevant for classification. In a neural network, feature values are probability values after applying softmax as an activation function on neuron outputs. Thus, our method posits that the joint probability between two single-valued features X and Y, is zero if their probabilities differ. In other words, mutual information between X and Y depends on their probability values, and a greater difference between these values suggests lower mutual dependence. The feature set relevancy maximization with an embedding-based approach in deep learning focuses on selecting or optimizing the most pertinent features for tasks like classification or prediction. This technique enables the model to create dense, informative representations of features in a high-dimensional space by leveraging embeddings. This, in turn, allows the model to concentrate on features that add the most value to its performance, enhancing overall task efficacy [11]. The filter approach excludes classifiers when selecting features, focusing instead on reducing redundancy. In this proposed method, a filter approach is applied in the first step, eliminating redundant features, followed by an embedded approach that selects the most relevant features from the refined set provided by the filter stage. This sequential process, termed cascaded feature selection, leverages mutual information by consecutively applying two complementary feature selection techniques to create an optimized feature set for classification. To simplify training complexity, we propose using a filter approach to minimize feature redundancy and an embedded approach to maximize feature relevance rather than relying on a single embedded method to address both challenges.

**Algorithm 1:** Feature selection technique.

Input: *n* = epochs count; m = group number for clustering; *k* = number of features to select
*F*= {fi | *i* = 1,2}: features from pre-trained CNNs ; *D* = Dataset considered
C={Ck | *k* = 1,2}: Target classes set
Output: F: Final set of features
1 Procedure for Filter Approach in Feature Selection
2 D_normalised = Normalised Data ∊ ℝ (samples x features) // Data preprocessed and Normalised //
3 Feature Correlation = Calculate correlation(D_normalised) Feature Correlation ∊ ℝ (features x features) //Calculation of Feature Correlation //
4 *S* = RankFeatures(D_normalized, y)
5 *F*= Remove the Redundant features (Redundant features identified, S, k) F ∊ {0, 1}features // Rank features and remove the Redundant features//
6 Return F (selected Features)
7 Procedure for feature selection using Embedded Approach
8 for each *j* = 1 to n do // To calculate the probabilistic differences for feature F1,i and all classes C //
9 D ← Diff(P(F1,i),P(C)): C=set of classes //Choose the class which has the lowest D for feature F1, i //
10 C1 ← Select (C) //Choose the feature F1, i if class Cl is not equal to the target class Ck //
11 ∼F ← Select (F1,i,F1)
12 end

In the above algorithm, the Feature selection method using a filter approach and embedded approach is presented. Features are more relevant for classification if they are less mutually dependent. The above algorithm reduces the number of features making it easier for analysis and visualization. In the initial step of feature selection using filter approach, Data is normalized for a mean value of zero and a variance value of unity. Then, the Correlation matrix is calculated for the normalized Input data, and by applying some correlation threshold, we will identify the Redundant feature remove the redundant feature, and output the selected feature. The embedded approach selects the relevant features obtained from the filter approach to get the final features set for classification purposes.

## Method validation

**Fig. 2:**
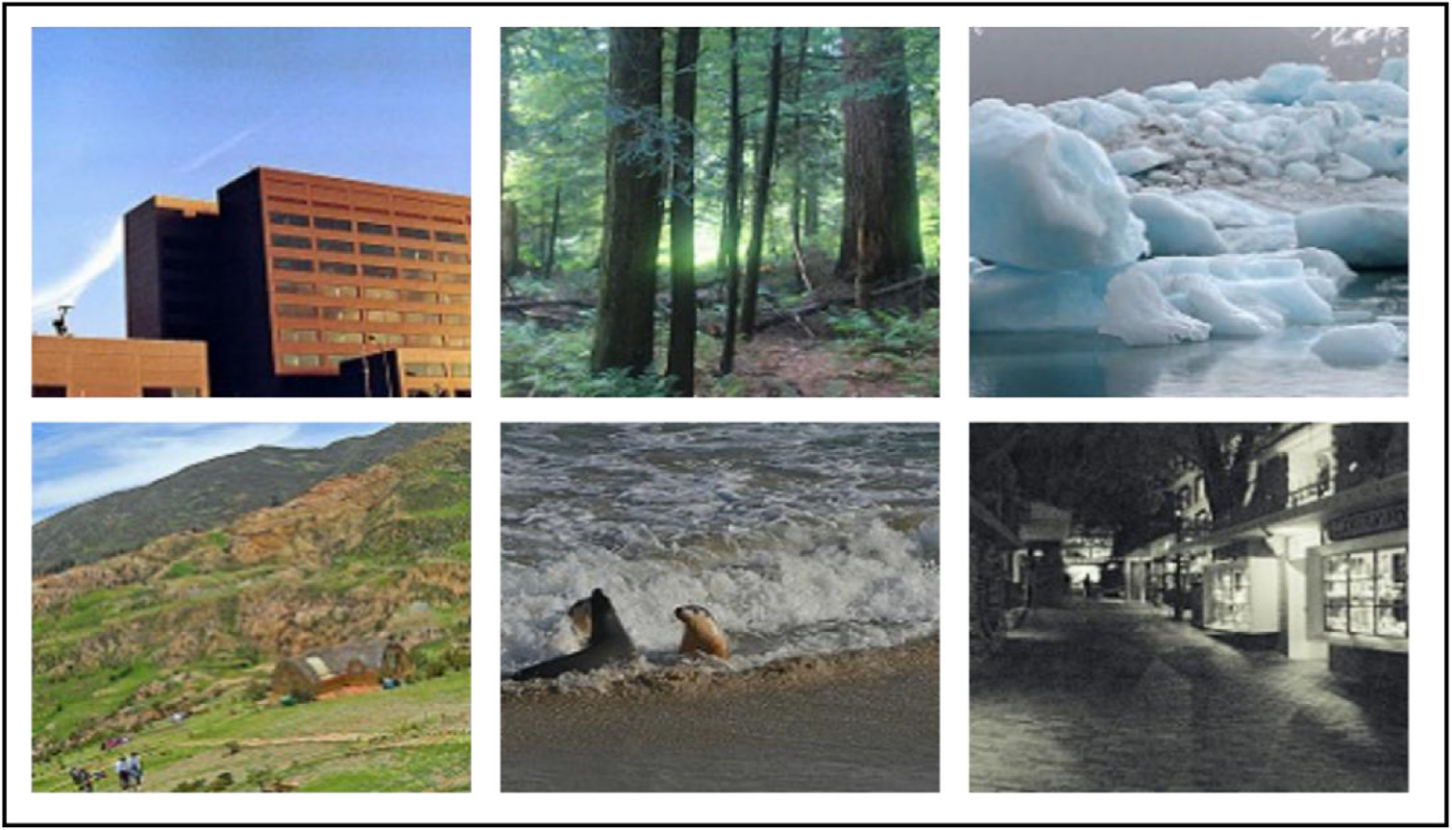
Sample images of six classes considered in the experimentation, buildings, forests, glaciers, mountains, sea, and streets.

we have considered a significant dataset to enable thorough model training and evaluation. The Scene Dataset used in our experimentation is obtained from Kaggle and consists of images categorized into six classes: buildings, forest, glacier, mountain, sea, and street. The Scene Classification dataset comprises approximately 25,000 images and covers various natural scene images worldwide. The main aim of the dataset is to classify scene images into multiple categories based on their characteristics. In our experimentation, we considered a classification problem that belongs into 6 class classifications where an image is expected to be categorized for one of the above-scene classes. In our experimentation, we selected 500 images belonging to each class for 3000 images. It is crucial to highlight that the Dataset has undergone data preprocessing steps, such as resizing, normalization, and data augmentation. These steps have been taken to ensure the quality and diversity of the dataset during model training and evaluation. The Input images are split into two subsets: training and testing. Around 80 % of the scene images are considered for training the network to learn about the visual features in the scene images. 20 % of images are constituted in a testing dataset to evaluate model scene recognition capabilities and performance. Images in the scene dataset have been resized to a size of 200 × 200. Fig. 2 shows the sample images from the dataset considered for scene classification. Details of the images included in each class are Buildings: This class comprises images of artificial buildings, such as houses, skyscrapers, and other structures. Forest: This class includes images of natural forest scenes with trees, vegetation, and landscapes. Glacier: This class contains images of glaciers, large masses of ice formed from snow accumulating over time. Mountain: This class comprises images of mountains and large landforms rising above the surrounding landscape. Sea: This class includes images of seas, oceans, or other bodies of saltwater. Street: This class comprises images of urban street scenes with buildings, roads, vehicles, and pedestrians.

The Dataset exhibits an equal number of images within each class while offering diverse scenes of various environments. The images are in RGB format and have varying resolutions, ranging from 256 × 256 to 1024 × 1024 pixels. It is crucial to highlight that the Dataset has undergone data preprocessing steps, such as resizing, normalization, and data augmentation. It has been applied to ensure the quality and diversity of the Dataset during model training and evaluation. Our deep learning model discussed above employed Convolution pooling and addition layers. The model was trained using the Adam optimizer using specific hyperparameters chosen, as shown in Table 2. Standard evaluation metrics, such as accuracy, precision, recall, and F1 score, are used to evaluate the model's performance.The metrics are defined as follows.

$$Accuracy=\;\frac{TP+TN}{TP+TN+FP+FN}$$

Where TP is True Positive TN is True Negative FP is False Positive FN is False Negative

$$Precision\;=\;\frac{TP}{TP+SP}$$

$$Recall\;=\;\frac{TP}{TP+FN}$$

$$F1\;Score\;=\;2\;X\;\frac{precision\;XRecall}{Precision\;+\;Recall}$$

Images in the Scene classification dataset are resized to a resolution of 200 × 200 pixels to ensure the consistent evaluation of the model's performance. Each image in the dataset is labeled properly with it's respective classes.

We have employed the Softmax Activation layer as the last layer in the proposed model. This layer transforms the Output from the previous layer to the probability distributions where each of the Scene classes has been assigned with different probabilities. The class with the highest probability is the predicted Scene. Here, we have employed a categorical cross-entropy loss function, which predicts the loss and indicates the dissimilarity between the expected class and true class. During training, the model learns to update the weights and bias values to get good prediction. Metrics such as accuracy, precision, recall, and F1 score are used to assess the model's capability to classify scenes into their respective classes. In our experimentation, we have also worked on an AID dataset. Xia et al. presented an AID Image dataset, a valuable resource with various diversified scene categorizations. The dataset comprises 30 different classes and 200 to 400 images, all of which are uniformly sized at 600 × 600 pixels, with a spatial resolution that varies from 0.5 to 8 m per pixel. These Images are extracted under various imaging conditions, seasons, and intra-class variations. In our experimentation, approximately 200 images of each class are considered, with 80 % of the images considered for training and 20 % for testing.

**Fig. 3:**
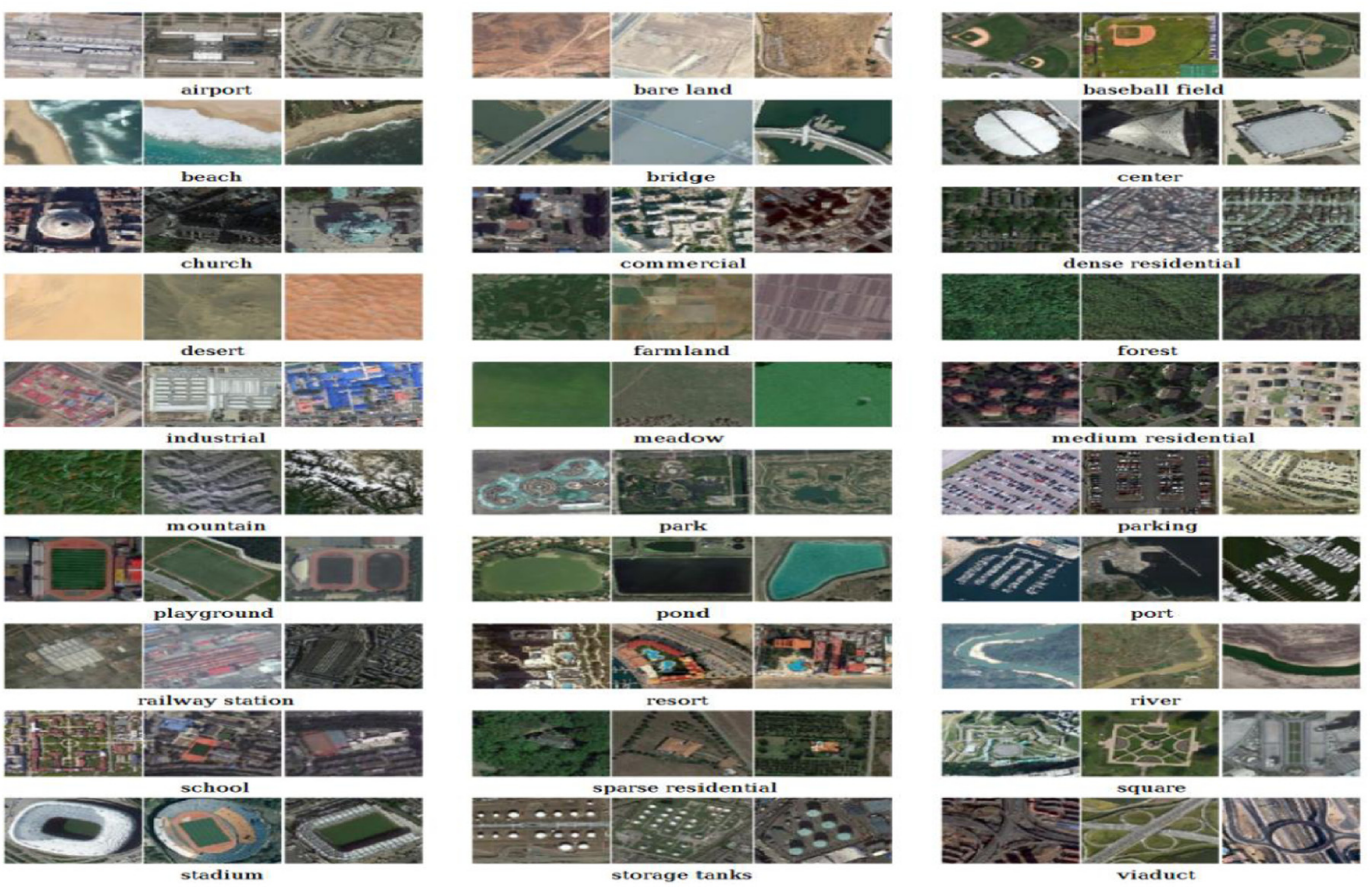
Sample images of AID dataset.

Fig 3 shows the AID dataset Image samples, which exhibit larger intra-class variations and lesser inter-class dissimilarity. The dataset has 10,000 images belonging to 30 different classes. Images in the AID dataset are sourced from Google Earth Imagery, encompassing various categories of Images collected from other countries.

## Results and discussions

In our experimentation, we have considered Scene Images belonging to 6 different classes. The dataset consists of 3000 Images, with 2400 images considered in the training dataset, and the remaining 600 images are placed in the testing dataset. Fig. 3 shows the output obtained during our implementation of the Training process. The response shown below represents that the model's accuracy increases with an increase in the number of epochs. The curves obtained illustrate the performance of the proposed model. The experiments were conducted on a system featuring an Intel Core i7 processor running at 2.4 GHz, 24 MB of RAM, and an NVIDIA GeForce RTX 3080 GPU with 16 MB of memory. In Fig. 3, The Accuracy and loss curves for the proposed model are plotted. At the outset, it is observed from the plot that the accuracy value increases and the loss value decreases. During the initial stages, it is observed that the accuracy value is 20 %, and an increase in the number of epochs increases the accuracy value to 100 %. The higher accuracy value signifies the model's proficiency in classifying the images.The loss curve is an indication of the model's optimization process. The loss curve shows that during the initial stage of training, the loss value is 3 % as the number of epochs increases, the loss value gradually converges to zero, representing the state of stability and enhancement in the scene classification task. It is noted that this remarkable training and validation performance was achieved efficiently.

**Fig. 4:**
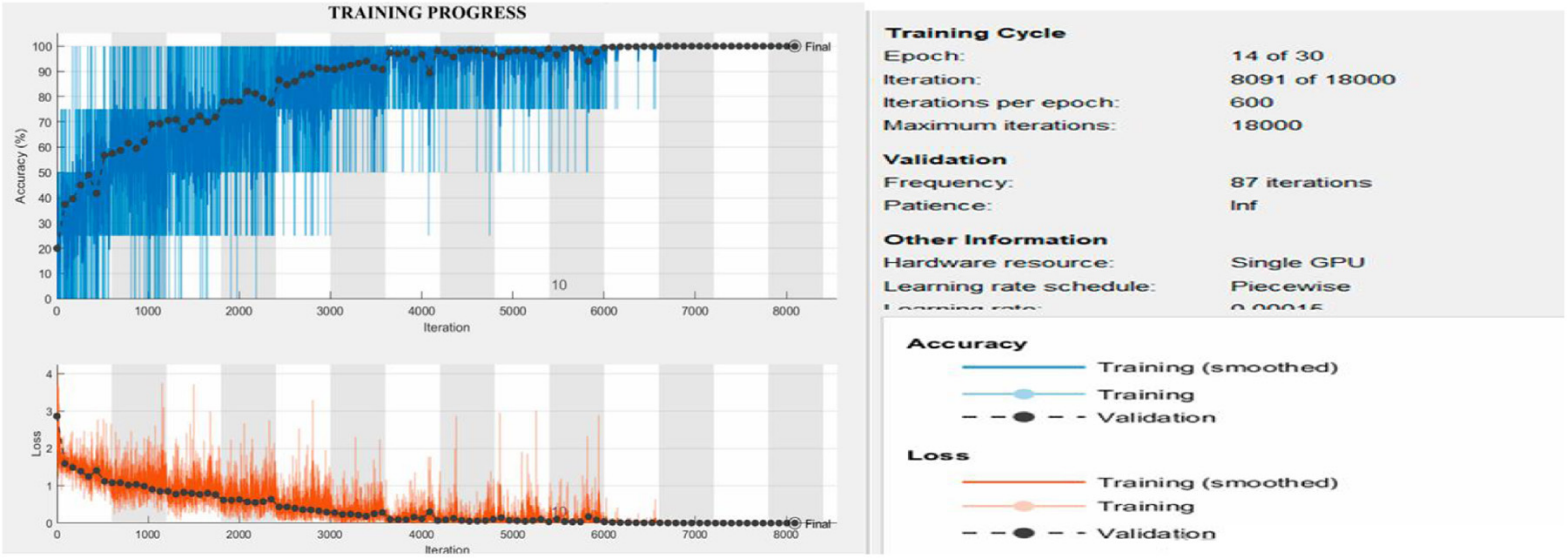
Training and validation progress of our method on the scene classification dataset.

Our deep learning model consists of Convolution layers pooling layers and was trained using Adam optimizer. The model involves the novel techniques of multimodal training and Feature selection employed to increase the efficiency of the model. Fig. 4 shows the model's capability to classify the Scene Images in the Dataset. Fig. 4 Shows the confusion matrix obtained by our proposed scene classification model.

**Fig. 5:**
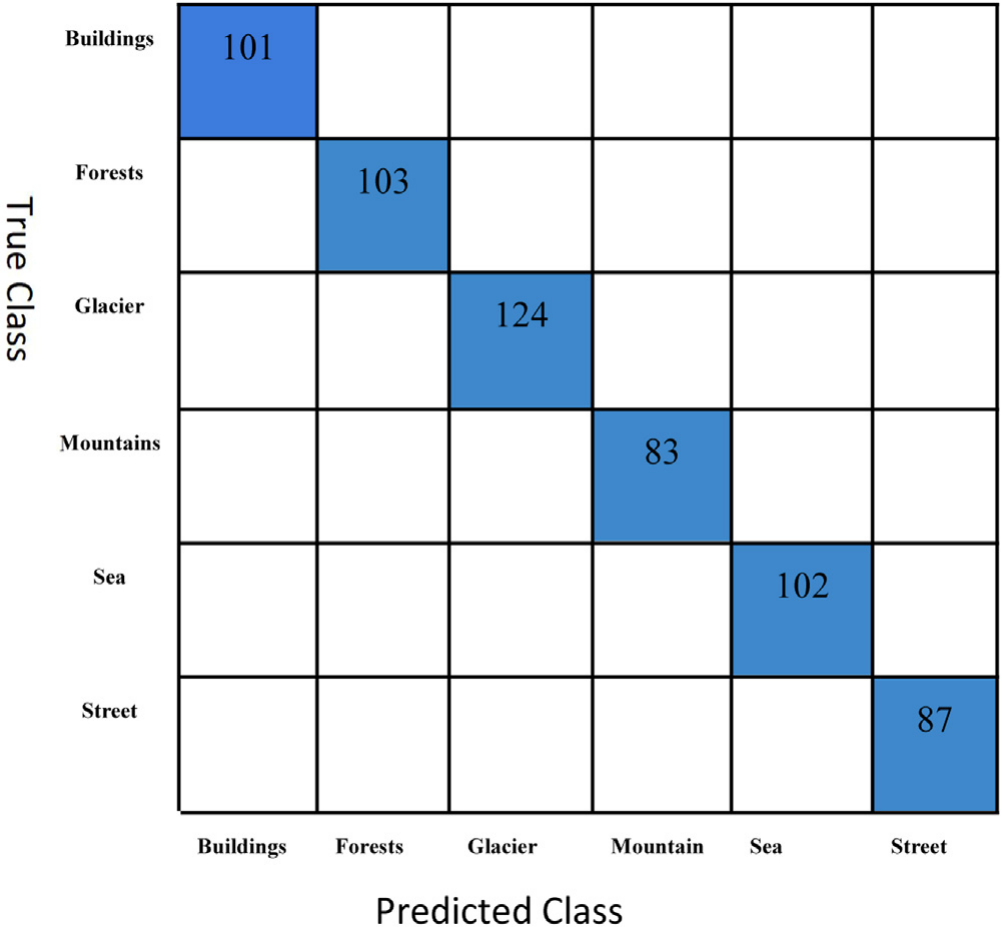
Confusion matrix on the scene classification dataset.

Fig. 5 illustrates the performance of our scene recognition model on the testing dataset, which includes six distinct scene classes: Buildings, Forests, Glaciers, Mountains, Sea, and Streets. The model performed well, accurately classifying 101 images in the Buildings category. Similarly, 103 photos from the Forest class were correctly identified. The Glacier class achieved perfect performance, with all 124 samples accurately classified. The model exhibited exceptional accuracy for the Mountains class, correctly identifying all 83 samples. The sea class was also precisely classified, and all 102 samples were accurately recognized. In the Street class, the model showed excellent results, correctly classifying 87 out of 87 samples.

**Fig. 6:**
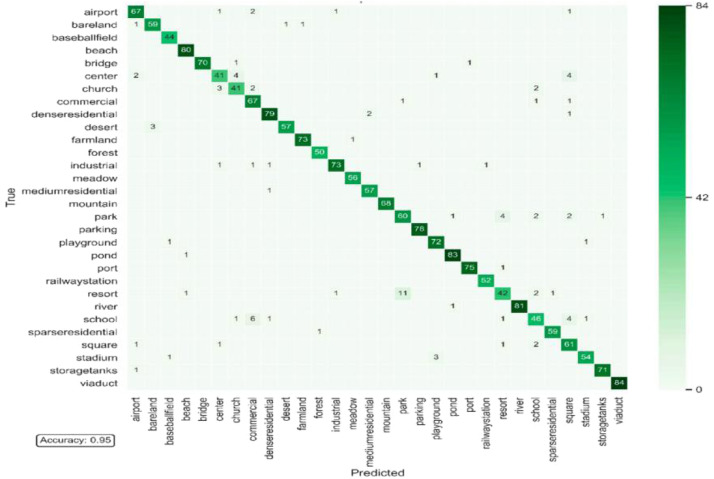
Confusion matrix obtained for AID dataset.

Fig. 6 shows the confusion matrix obtained for the AID Dataset. Our model performed well in recognizing the images. The results have demonstrated good performance in identifying the Scene classes. This approach is entirely distinct from the traditional classification methods of Scene Data. This approach enhances the ability of the model to classify the data. The experiment is conducted on the Scene dataset [13]. Our proposed model has improved the results of classifying the Scene Image data. Table 3 summarizes the results, and our model has achieved outstanding results.

**Table 3:** Performance of our model on the Scene Classification dataset.

Data	Accuracy	Precision	Recall	F1-Score
Scene Data [13]	99.90 %	99.90 %	99.90 %	99.90 %
AID Dataset [12]	95.52 %	95.50 %	95.50 %	95.50 %

The table shows that our approach demonstrates superior performance. Finally, our proposed methodology can combine CNN with a Multimodal deep learning network and a feature selection technique to remove the redundant features and select the relevant features, providing a good solution for scene recognition tasks. We aim to highlight that the extraordinary performance of our model may be attributed to our creative approach, rigorous methodology, and the integration of feature selection based on a Multimodal deep learning network. The proposed feature selection method is designed to excel in classification tasks across diverse categories, ensuring robustness and efficacy in real-world applications.

## Limitations

Not applicable.

## Ethics statements

The Scene Dataset used is freely downloadable. This research did not involve human subjects or animal models, and social media data was not used.

## CRediT author statement

**Sumathi K:** Responsible for project administration, conceptualization, methodology, data curation, investigation, validation, supervision, visualization, drafting the original manuscript, and reviewing and editing the final document.

**Dr. Pramod Kumar S:** Contributed to project administration, investigation, data curation, supervision, and reviewing and editing of the manuscript.

**Dr. H R Mahadevaswamy & Ujwala B S:** Involved in project administration, conceptualization, methodology, investigation, and manuscript review and editing

## Declaration of competing interest

The authors declare that they have no known competing financial interests or personal relationships that could have appeared to influence the work reported in this paper.

## Data Availability

Data will be made available on request.
